# Comparison and selection of probiotic *Lactobacillus* from human intestinal tract and traditional fermented food in vitro via PCA, unsupervised clustering algorithm, and heat‐map analysis

**DOI:** 10.1002/fsn3.3018

**Published:** 2022-08-22

**Authors:** Longfei Zhang, Hengxian Qu, Xiaoxiao Liu, Qiming Li, Yang Liu, Wenqiong Wang, Dawei Chen, Lixia Xiao, Ruixia Gu

**Affiliations:** ^1^ College of Food Science and Engineering Yangzhou University Yangzhou China; ^2^ Jiangsu Key Laboratory of Dairy Biotechnology and Safety Control Yangzhou China; ^3^ Jiangsu Dairy Biotechnology Engineering Research Center, Kang Yuan Dairy Co. Ltd Yangzhou University Yangzhou China; ^4^ New Hope Group Chengdu China

**Keywords:** heat‐map analysis, human and nonhuman resources, *Lactobacillus*, PCA, physiological characteristics, unsupervised clustering

## Abstract

Traditional fermented products and human intestines are rich sources of *Lactobacillus* strains which may have remarkable probiotic properties. In the present study, the probiotic properties of 40 *Lactobacillus* strains isolated from intestinal tracts of longevity population and traditional fermented food in China were determined, including the survival rates in simulated gastric acid and bile salt, aggregation, hydrophobicity, adhesion rate, antioxidant ability (ferric reducing antioxidant power), and antimicrobial ability. The differences between human strains and nonhuman strains were compared via *t*‐test and principal component analysis (PCA). The significant differences were found in the survival rate at 0.3% bile salt, adhesion ability of the strains, and antioxidant ability of the fermentation broth (*p* < .05). The results of PCA showed that the first principal component (PC1) score of human strains was significantly higher than that of nonhuman strains (*p* < .01). And some probiotic *Lactobacillus* were selected for further application based on the unsupervised clustering algorithm, heat‐map analysis, and K‐means algorithm. Four strains, CS128, CS39, CS01, and CS1301, along with *Lactobacillus rhamnosus* GG (LGG) were divided into cluster I. The four strains, all isolated from human tracts, have been selected. Thus, human *Lactobacillus* has better probiotic potential and application prospects than strains from the nonhuman source. PCA, the unsupervised clustering algorithm, and heat‐map analysis can be used to analyze and select *Lactobacillus* visually and effectively.

## Graphical abstract

Our findings propose that we can select *Lactobacillus* with better probiotic properties from the human source more easily. Principal component analysis (PCA), the unsupervised clustering algorithm, and heat‐map analysis can be used to analyze and select *Lactobacillus* visually and effectively.

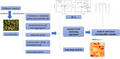

## 1 INTRODUCTION

Probiotics are live microorganisms that provide health benefits to the host via changing the gut microbiota when administered in an adequate amount (Liu et al., 2007; Park et al., 2002). *Lactobacillus* comprise an important group of fermentative bacteria widely used for the production of fermented foods in both household and industry as starter cultures and are also a part of normal intestinal microflora. The study on the probiotic spectrum of *Lactobacillus*, including nutritional, physiological, and antibacterial effects, pushes the development of *Lactobacillus* for humans and animals (Boricha et al., 2019). For the application of probiotic *Lactobacillus*, it is recommended to value some properties such as acid and bile tolerance, antimicrobial and antioxidant activity, cell surface hydrophobicity, adhesion ability to epithelial cells, and so on (Mallappa et al., 2019).

In order to develop probiotic *Lactobacillus*, researchers obtained *Lactobacillus* from human sources, such as feces (Wang et al., 2020), breast milk (Kang et al., 2020), mouth (Bazireh et al., 2020), and natural fermented food sources, such as fermented milk (Vasiee et al., 2020), pickles (Qian et al., 2018), and cheese (Domingos‐Lopes et al., 2020). The previous studies have proved that *Lactobacillus* screened from human and natural fermented food both had certain probiotic characteristics (Shokryazdan et al., 2017). In addition, many studies have shown that the strains will undergo a variety of changes such as growth kinetic, enzyme production, the expression of specific proteins, and so on due to changes in their growth environment. (Maresca et al., 2018; Mbye et al., 2020; Wu et al., 2011). Therefore, *Lactobacillus* strains from different sources may have different abilities due to different growth environments.

Unsupervised clustering algorithms and heat‐map analysis have been proposed as alternative approaches for reproducible clustering of different datasets (Panahi, Mohammadi, et al., 2019) and this approach recognizes commonalities within the information. And it identifies anomalous data focuses that do not fit into either bunch (Panahi et al., 2020). This feature allows us to divide a large number of experimental strains into different clusters rather than comparing each property individually. In many cases, the probiotic properties of a candidate strain are not always outstanding. When screening probiotic strains via conventional methods, strains with good probiotic properties but slightly poorer viability may be missed. This feature allows us to take all properties into account and make comparisons. Principal component analysis (PCA) is a well‐known method for dimensionality reduction and data representation (Wang, Gao, et al., 2018) and has been considered crucial for the selection of parameters that elucidates the differentiation of strains by a small number of linear combinations of the variabilities among assays (Sharma et al., 2019). The lack of a high probability of target sources usually makes the screening process more time‐consuming and labor‐consuming (Agarbati et al., 2021; Boricha et al., 2019; Nami et al., 2020; Shen et al., 2011). In this regard, the focus of the present study was to determine and compare the properties of *Lactobacillus* from human and natural fermented food, select some probiotic *Lacto*bacillus visually, and find out which is the source of high probability of obtaining high‐quality probiotic *Lactobacillus*.

## 2 MATERIALS AND METHODS

### 2.1 Isolation and identification

Fermented dairy samples and vegetables were purchased freshly from local market in Yunnan Province and in Jiangsu Province separately. Human source strains were isolated from intestinal tracts of longevity population in Guangxi province. As much as 0.1 g sample was taken and cultured in 10 ml in Man Rogosa Sharpe (MRS) medium at 37°C for 24 h. The loopful of enriched culture was streaked on MRS agar and incubated at 37°C. The colonies with morphological characteristics of lactic acid bacteria (LAB) were subcultured until purified on MRS agar (Boricha et al., 2019). Finally, the strains were characterized by morphological examination using light microscopy and using Gram stain tests.

The strains were further identified on the basis of 16S rDNA sequence analysis using primers of 27F (5′‐AGAGTTTGATCCTGGCTCAG‐3′) and 1492R (5′‐TACGGCTACCTTGTTACGACTT‐3′) (Kang et al., 2020). For identification of *Lactobacillus plantarum* (Kostelac et al., 2021), *Lactobacillus paraplantarum*, and *Lactobacillus pentosus*, the recA loci (Torriani et al., 2001) were amplified and sequenced with primers LbrecA‐f (5′‐TTGGCTGATGCACGGAAA‐3′) and LbrecA‐r (5′‐GCGAGGATTATACCGAAAACATTCAT‐3′) designed in this study. The PCR products were sequenced by Sangon Biotech Co., Ltd. (Shanghai, China) and compared with the National Center for Biotechnology Information (NCBI) BLAST sequence database (https://blast.ncbi.nlm.nih.gov/Blast.cgi) to identify the strains (Ding et al., 2017).

### 2.2 Microorganisms and culture conditions

A total of 40 *Lactobacillus* strains isolated from traditional fermented dairy samples, fermented vegetables, and human intestine tracts were cultured in MRS medium at 37°C for 24 h. *Lactobacillus rhamnosus* GG (LGG) obtained from the Shanghai Bioresources Collection Center was cultured under the same condition and used as the reference strain.

The indicator strains *Bacillus subtilis* CICC10012, *Escherichia coli* CICC10899, and *Salmonella* WX29 obtained from China Center of Industrial Culture Collection (CICC) were cultured in Luria‐Bertani (LB) medium at 37°C for 12 h.

### 2.3 Cell lines and culture condition

The intestinal Caco‐2 cell line obtained from the Institute of Cell Research, Chinese Academy of Sciences was cultured in high‐glucose minimum essential medium (MEM; Gibco) supplemented with 20% (v/v) inactivated (56°C, 30 min) fetal bovine serum (FBS) (Gibco) and 100 U of penicillin‐streptomycin (Gibco). The cells were incubated at 37°C with 5% CO_2_ (Damodharan et al., 2015).

### 2.4 Tolerance to simulated gastric acid and bile salt

The tolerance to simulated gastric acid and bile salt was determined by the previous method (Leandro et al., 2021) with modifications. As much as 0.3 g pepsin (Sigma) and 0.5 g NaCl (Sinopharm) were dissolved in 100 ml deionized water. As much as 1 mol/L HCl (Sinopharm) was added to set its pH at 3.0 and 2.0. The overnight‐cultured (37°C, 24 h) strains were harvested by centrifugation (4°C, 6000 g, 5 min). The strains were washed three times with phosphate‐buffered saline (PBS; pH 7.2) and then were added into simulated gastric acid.

The washed strains were added into the MRS medium containing 0.3% and 0.1% (w/v) bile salt (Solarbio). The number of viable bacteria at 0 and 3 h was checked. The tolerance of the strains to simulated gastric acid and bile salt was determined as follows:

$$\text{The survival rate}\;\left(\%\right)=\frac{\text{the number of viable bacteria}\;\mathrm{at}\;3\;h}{\text{the number of viable bacteria}\;\mathrm{at}\;0\;h}.$$

### 2.5 Hydrophobicity and auto‐aggregation

The aggregation ability and cell surface hydrophobicity were determined by the previous method (Krausova et al., 2019) with some modifications. The strains were grown in MRS at 37°C for 24 h, harvested by centrifugation (4°C, 6000 g, 5 min), and washed twice with PBS (pH 7.2). For aggregation, the strains were resuspended in PBS (pH 7.2). The absorbance at 600 nm (OD_1_) was measured. After incubation at the room temperature for 6 h, the absorbance of the supernatant was measured again (OD_2_). The aggregation was calculated with the following equation:

$$\text{Aggregation}\;\left(\%\right)=\frac{\left({\mathrm{OD}}_{1}-{\mathrm{OD}}_{2}\right)}{\left({\mathrm{OD}}_{1}\right)}.$$

For hydrophobicity, the strains were harvested by centrifugation (4°C, 6000 g, 5 min), washed three times, and resuspended in PBS (pH 7.2) to obtain OD_600_ (A_1_) = 0.7 ± 0.02. Then, 4 ml solution and 0.4 ml n‐hexadecane (Merck) were mixed and incubated for 3 h at 37°C. The absorbance of the aqueous phase was read at 600 nm (A_2_). The value was calculated with the following equation:

$$\text{Hydrophobicity}\;\left(\%\right)=\frac{\left(A_{1}-A_{2}\right)}{A_{1}}.$$

### 2.6 Adhesion ability

The adhesion ability was determined by the fluorescent probe (Lee et al., 2004). *Lactobacillus* strains (10^10^ CFU/ml) and 50 μM carboxyfluorescein diacetate‐succinimidyl ester (cFDA‐SE; Beyotime) were mixed and incubated in water bath at 37°C in the dark for 20 min. The strains were centrifuged (4°C, 6000 g, 5 min) and washed with PBS (pH 7.2) three times. The percentage of fluorescence‐labeled live cells was 99.74% with the determination of the labeled strains.

The cultured Caco‐2 cells were digested and diluted with high‐glucose minimum essential medium (MEM) solution to a concentration of 5 × 10^5^ cells/ml. The 1 ml cell suspension was added into a 12‐well cell culture plate and cultured in a monolayer at 37°C with 5% CO_2_. One milliliter of the labeled strains (10^8^ CFU/ml) in PBS (pH 7.2) was added into the cells. After incubation at 37°C with 5% CO_2_ for 3 h, the cells were washed with PBS (pH 7.2) to remove the unadhered strains. The adhered strains were collected with 1 ml PBS (pH 7.2). Then the fluorescent intensity of the fluid was measured after adhesion using a fluorescence spectrophotometer (Shimadzu Corporation) with an excitation (EX) wavelength of 450 nm, an emission (EM) wavelength range starting at 470 nm and ending at 650 nm, and a slit width EX at 3 nm and EM at 5 nm. The adhesion ability was calculated with the following equation:

$$\text{Adhesion}\;\left(\%\right)=\frac{\text{fluorescence intensity of adhered cells}}{\text{total fluorescence intensity of cells}}.$$

### 2.7 Antimicrobial ability of fermented broth

The antimicrobial ability of *Lactobacillus* strains was determined using the agar well diffusion method (Hai et al., 2021). Briefly, 100 μl of cultured *Bacillus subtilis*, *Salmonella*, and *Escherichia coli* was poured and separated evenly on the LB agar plates. The holes of 9‐mm diameter were bored on a plate with a sterile hole punch. Then, 200 μl of fermentation broth (37°C, 24 h) was shaken and added to the hole. The plates were incubated at 37°C for 12 h and then the diameter of bacteriostatic zone was measured.

### 2.8 Antioxidant ability

The antioxidant ability of *Lactobacillus* strains and their fermentation broth was determined by using the antioxidant ability assay kit (Jiancheng, China). The fermentation broth (37°C, 24 h) was reprepared. The cultured strains were harvested by centrifugation (4°C, 6000 g, 5 min) and resuspended in PBS (pH 7.2). Different concentrations (0.15, 0.3, 0.6, 0.9, 1.2, and 1.5 mM) of FeSO_4_‐7H_2_O solutions were prepared with distilled water. Five microliters of different concentrations of the standard solution, the strains (10^8^CFU/ml), and the fermentation broth were added into 96‐well plates. Then, 180 μl of FRAP fluid was added. After incubation at 37°C for 3–5 min, the absorbance at 593 nm was measured. The standard curve of the concentration and absorbance values were plotted. The absorbance of the samples was determined and substituted into the curve. The antioxidant ability was shown as the concentration of FeSO_4_‐7H_2_O.

### 2.9 Statistical analysis

Data were presented as Box & Whisker's plot with 5%–95% percentile by using Origin 2018. Statistical differences in the results were analyzed by one‐way analysis of variance (ANOVA) and significant differences between different sources by *t*‐tests using IBM SPSS Statistics 22 at *p* < .05 for the determination of significance. Principal component analysis (PCA) and cluster analysis were performed to discriminate samples on the basis of probiotic properties by Origin 2018. The heat map was generated using Euclidean distance and complete linkage algorithm implemented in the Heat‐Map Dendrogram of Origin 2018 software. For the unsupervised clustering of samples, the K‐means algorithm was used in this study. This algorithm uses kernels to estimate the distance between objects and clusters (Panahi, Frahadian, et al., 2019).

## 3 RESULTS AND DISCUSSION

### 3.1 Isolation and identification of *Lactobacillius*

Forty strains isolated from human sources and nonhuman sources including pickles, spicy sauce, pickled cucumber, fan‐like cheese, and cheese were identified as *Lactobacillus* by the 16s rDNA sequence analysis (Table 1). These isolates were identified as *L. plantarum* (12 human isolates and 10 nonhuman isolates), *L. fermentans* (six human isolates and three nonhuman isolates), and *L. rhamnosus* (four human isolates and five nonhuman isolates).

**TABLE 1:** List of experimental strains

Strains	Origin	Species	Strains	Origin	Species
CS39	Human	*L. plantarum*	pA	Pickles	*L. plantarum*
CSS7	Human	*L. plantarum*	pm	Pickles	*L. plantarum*
CS11	Human	*L. plantarum*	pg	Pickles	*L. plantarum*
CS4	Human	*L. plantarum*	pk	Pickles	*L. plantarum*
CS5	Human	*L. plantarum*	x3	Spicy sauce	*L. plantarum*
CS1	Human	*L. plantarum*	p10	Pickles	*L. plantarum*
CS90	Human	*L. plantarum*	x7	Spicy sauce	*L. plantarum*
CS97	Human	*L. plantarum*	pd	Pickles	*L. plantarum*
CS113	Human	*L. plantarum*	h5	Pickled cucumber	*L. plantarum*
CS69	Human	*L. plantarum*	hc	Pickled cucumber	*L. plantarum*
CS128	Human	*L. plantarum*	B02	Fan‐like cheese	*L. fermentans*
CS154	Human	*L. plantarum*	xN	Spicy sauce	*L. fermentans*
CS56	Human	*L. fermentans*	p8	Pickles	*L. fermentans*
CS57	Human	*L. fermentans*	B19	Fan‐like cheese	*L. rhamnosus*
CS54	Human	*L. fermentans*	g7	Cheese	*L. rhamnosus*
CS07	Human	*L. fermentans*	g10	Cheese	*L. rhamnosus*
CS08	Human	*L. fermentans*	g13	Cheese	*L. rhamnosus*
CS10	Human	*L. fermentans*	B22	Fan‐like cheese	*L. rhamnosus*
CS01	Human	*L. rhamnosus*	CS1301	Human	*L. rhamnosus*
CS108	Human	*L. rhamnosus*	CSLYO	Human	*L. rhamnosus*

### 3.2 Tolerance to simulated gastric acid and bile salt

**FIGURE 1:**
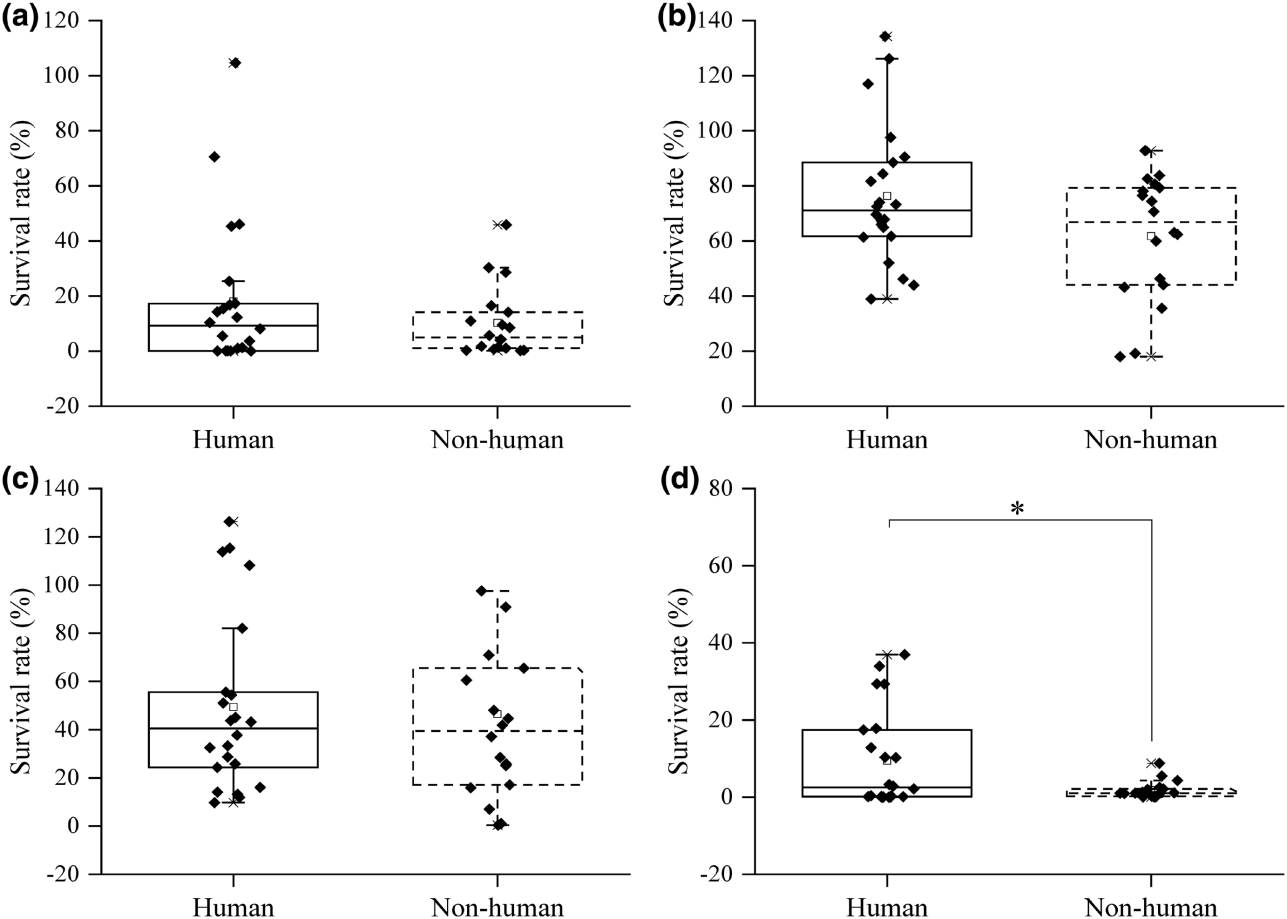
Comparison of the survival rate in simulated gastric acid and bile salt of strains from different sources. a: pH 2.0, b: pH 3.0, c: 0.1% bile salt, and d: 0.3% bile salt. All the data were tested three times and averaged. * represents significance (*p* < .05).

The tolerance to simulated gastric acid and bile salt of the strains from different sources was determined and the result of comparison is shown in Figure 1. At pH 2.0, the highest survival rate, average survival rate, and the survival rate of the top 25% of human strains were higher than those of nonhuman strains, but there was no significant difference (*p* > .05) (Figure 1a). However, the survival rate of 19 human strains (86.36%) was more than 50%, while that of nonhuman *Lactobacillius* was only 12 (66.67%) at pH 3.0. The maximum survival rate, minimum survival rate, the top 25%, the later 25%, and average survival rate of human source were higher than those of a nonhuman source but there was no significant difference (*p* > .05) (Figure 1b).

For 0.1% bile salt, the survival rate of human strains over 50% was eight (36.36%) and that of nonhuman strains was six (33.33%). There was no significant difference (*p* > .05; Figure 1c). For 0.3% bile salt, the survival rate of nonhuman *Lactobacillus* was centralized, and all of them were lower than 10%. There was a significant difference in survival rate between human and nonhuman strains (*p* < .05; Figure 1d). Human *Lactobacillius* has better tolerance to 0.3% bile salt than nonhuman *Lactobacillius*.

### 3.3 Adhesion ability in vitro

**FIGURE 2:**
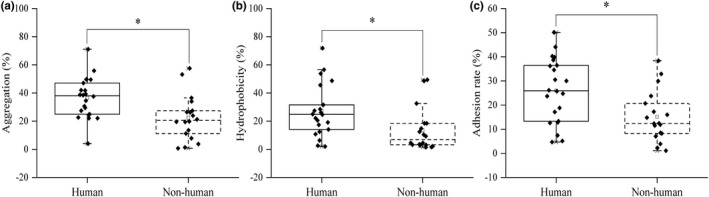
Comparison of the adhesion ability of strains from different sources. a: Aggregation. b: Hydrophobicity. c: Adhesion ability. All the data were tested three times and averaged. * represents significance (*p* < .05).

Auto‐aggregation represents the ability to form biofilms. The ability of aggregation has been proved to be related to the ability of bacteria to adhere to intestinal mucosa and epithelial cells (Ferreira et al., 2011). The auto‐aggregation ability of *Lactobacillus* is shown in Figure 2a. The auto‐aggregation ability of human strains ranged from 4.07% to 71.11%, while that of nonhuman strains ranged from 0.73% to 57.48%. The auto‐aggregation ability of human strains was significantly higher than that of nonhuman strains (*p* < .05).

The initial contact between an organism and the host cells was contributed by hydrophobicity, a measure of the relative tendency of a substance to prefer a nonaqueous environment (Shobharani & Agrawal, 2011). The hydrophobicity of human strains ranged from 2.02% to 71.81%, while that of nonhuman strains ranged from 1.48% to 49.38% (Figure 2b). The hydrophobicity of human strains was significantly higher than that of nonhuman strains (*p* < .05).

The adhesion ability of human strains to Caco‐2 cells was 4.69%–50.11%, while that of nonhuman strains was 1.08%–32.89% (Figure 2c). The adhesion ability of human strains was significantly higher than that of nonhuman strains (*p* < .05) via *t* test.

### 3.4 Antimicrobial ability

**FIGURE 3:**
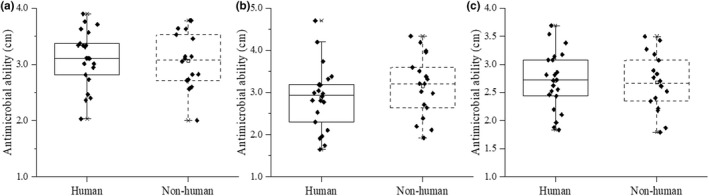
Comparison of the antimicrobial ability of strains from different sources. a: *Escherichia coli*. b: *Salmonella*. c: *Bacillus subtilis*. All the data were tested three times and averaged.

The antimicrobial ability of the fermentation broth of strains from different sources is shown in Figure 3. The inhibition zone diameter of human *Lactobacillus* fermentation broth against *Escherichia coli* ranged from 2.36 to 3.76 cm, while that of nonhuman *Lactobacillus* fermentation ranged from 2.00 to 3.78 cm. The inhibition zone diameter of human strains fermentation broth against *Salmonella* was 1.74–4.70 cm, and the inhibition zone diameter of nonhuman *Lactobacillus* fermentation was 1.92–4.34 cm. The inhibition zone size of human strains fermentation broth against *Bacillus subtilis* was 1.83–3.68 cm, and that of nonhuman strains fermentation broth was 1.79–3.49 cm. The inhibition zone size of different strains fermentation broth was different, there was no significant difference in the antimicrobial ability of the strains from different sources (*p* > .05).

### 3.5 Antioxidant ability

**FIGURE 4:**
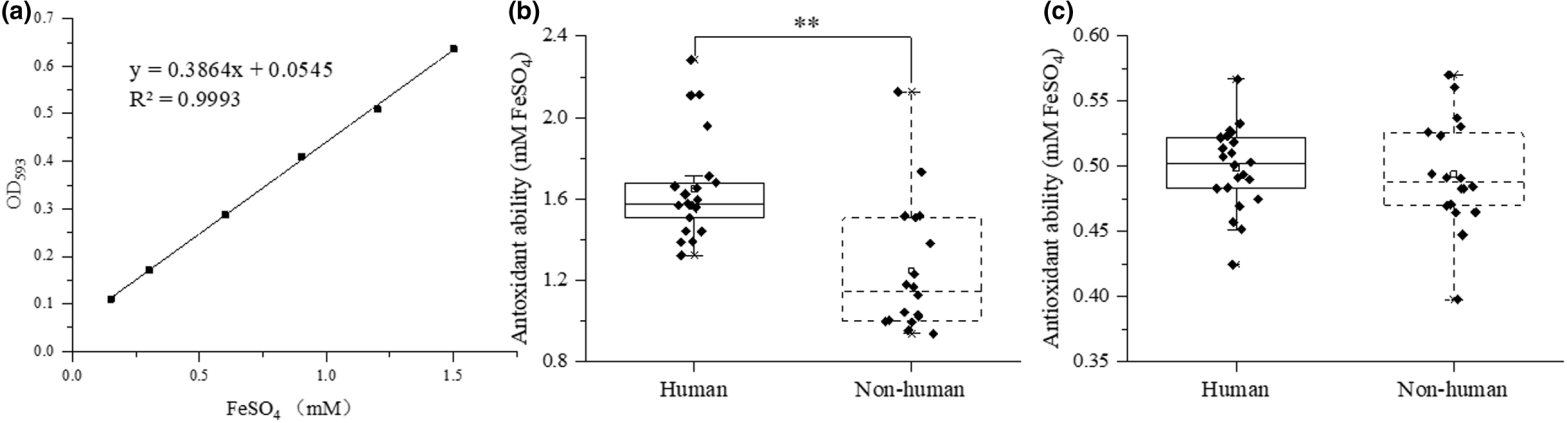
Comparison of the antioxidant ability of strains from different sources. a: The standard curve. b: Fermentation broth. c: Strain. All the data were tested three times and averaged. * represents significance (*p* < .05).

The standard curve of the concentration and absorbance values and the comparison of antioxidant capacity is shown in Figure 4. The standard curve is *y* = 0.3864*x* + 0.0545 (*R* ^2^ = 0.9993) (Figure 4a). The antioxidant capacity of human strains fermentation broth was 1.32 mmol FeSO_4_–2.28 mmol FeSO_4_, of which 17 strains were more than 1.5 mmol FeSO_4_, accounting for 77.27%. The antioxidant capacity of nonhuman fermentation broth was 0.94 mmol FeSO_4_–2.13 mmol FeSO_4_, of which five strains were more than 1.5 mmol FeSO_4_, accounting for 27.78%. The antioxidant capacity of human strains fermentation broth was significantly higher than that of nonhuman fermentation broth (*p* < .05; Figure 4b), indicating that antioxidant capacity of fermentation broth was higher than that of the strain itself.

The comparison of antioxidant capacity of different strains is shown in Figure 4c. The antioxidant capacity of human strains was 0.42 mmol FeSO_4_–0.57 mmol FeSO_4_, while that of nonhuman strains was 0.40 mmol FeSO_4_–0.57 mmol FeSO_4_. There was no significant difference in the antioxidant capacity of the strains (*p* > .05).

### 3.6 Principal component analysis of *Lactobacillus* characteristics

**FIGURE 5:**
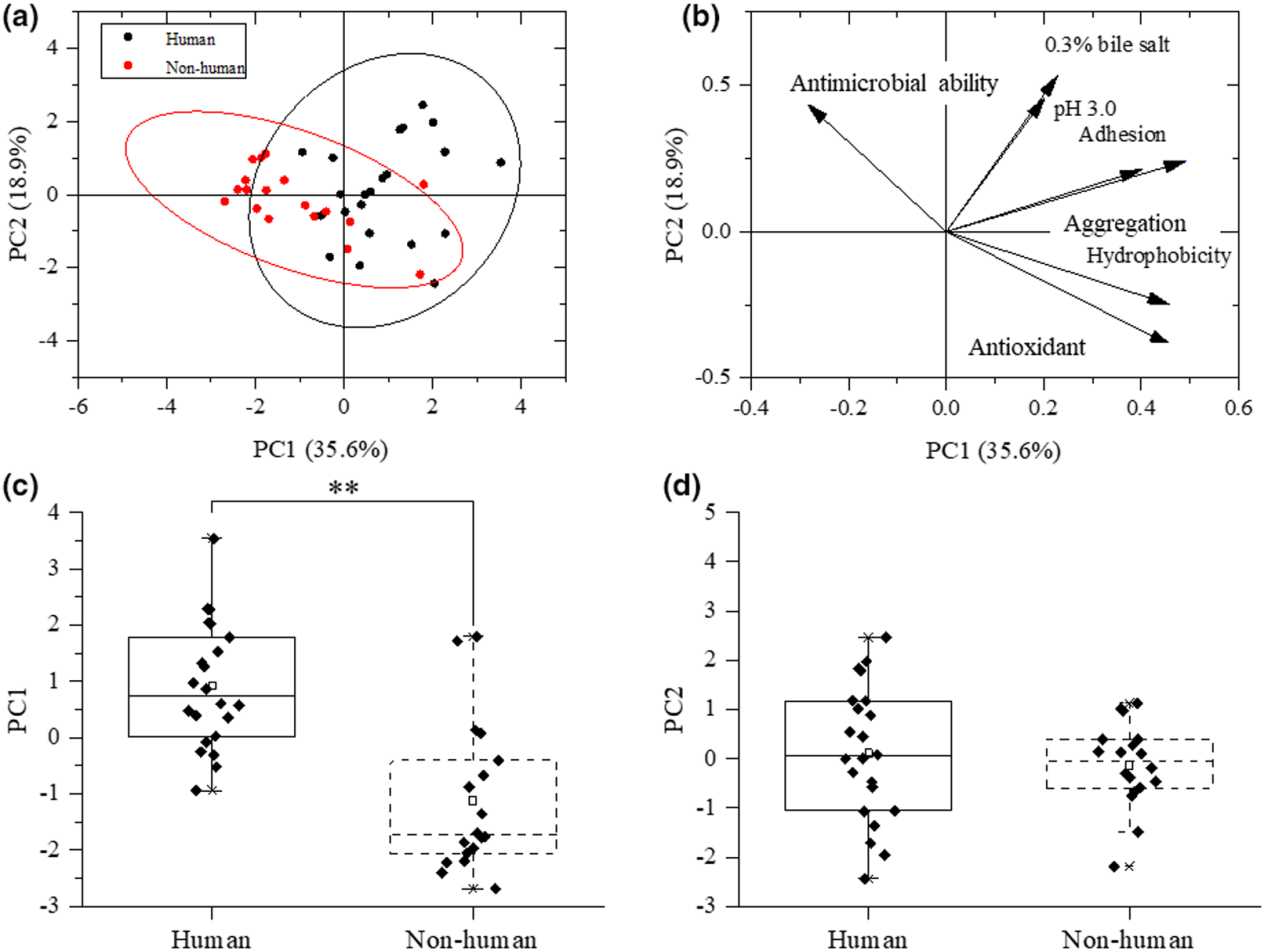
Probiotic properties of the strains from different sources via principal component analysis (PCA). a: Score plot. b: Loading plot. c: First principal component (PC1) score of strains from different sources. d: Second principal component (PC2) score of strains from different sources. Differences between sources were calculated via *t*‐test. ** represents significant differences (*p* < .01).

Principal component analysis (PCA) was used to analyze the tolerance of human and nonhuman strains to 0.3% bile salt, pH 3.0 simulated gastric acid, adhesion ability, antioxidant ability of fermentation broth, and antibacterial ability to *Escherichia coli* (Figure 5). The first principal component (PC1) explained 35.6% of the total change. The second principal component (PC2) explained 18.9% of the total change. Except that the antibacterial activity was in the negative region of PC1, the other characteristics were in the positive region. Seventeen human strains (72.27%) and fourteen nonhuman strains (77.78%) were separated in the positive and negative regions of PC1. The PC1 score of *Lactobacillus* from different sources showed that the human *Lactobacillus* strains had better properties than the nonhuman *Lactobacillus* strains (*p* < .01). There was no significant difference in PC2 score (*p* > .05).

### 3.7 Probiotic isolates selection

**FIGURE 6:**
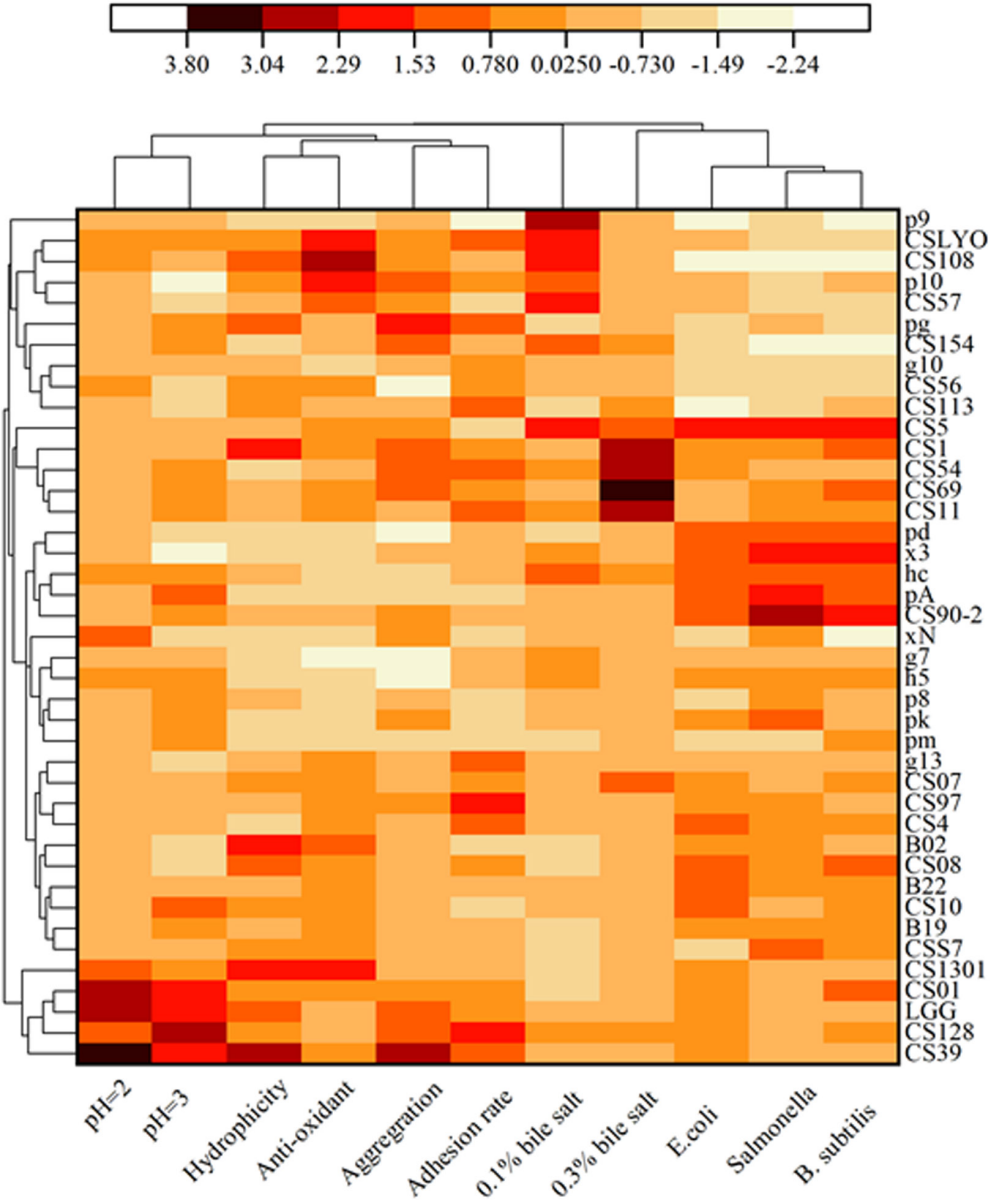
The heat‐map and cluster analysis.

In order to distinguish appropriate candidate probiotic isolates for future studies and figure out which was a better source for probiotics isolation, the relationship between the probiotic isolates was surveyed based on the important probiotic properties. The results obtained from the heat‐map analysis of the candidate isolates are shown in Figure 6. It was clear that 40 *Lactobacillus* isolates and reference strain LGG were clustered into two main clusters and four subclusters. Four strains from human sources including CS39, CS128, CS01, CS1301 and LGG belonged to cluster I, while all 18 strains from nonhuman sources belong to cluster II via hierarchical agglomerative clustering. Cluster analysis based on the K‐means algorithm, belonging to partitioning clustering, grouped isolates into five distinct clusters and the results are shown in Table 2. More interestingly, cluster analysis of isolates based on unsupervised algorithms also clustered CS39, CS128, CS01, CS1301 and LGG in the same cluster, highlighting the efficiency and feasibility of applied approaches in the detection of probiotic isolates based on the phenotypic data.

**TABLE 2:** Cluster analysis based on K‐means algorithm

I	II	III	IV	V
CS39	CS154	CSS7	CS97	CS11
CS128	CS57	CS4	CS113	CS5
LGG	CS108	CS90	CS56	CS1
CS1301	CSLYO	CS10	pg	CS69
CS01	p10	pA	g10	CS54
	p9	pm	g13	CS07
		pk		CS08
		x3		
		pd		
		h5		
		hc		
		B02		
		xN		
		p8		
		B19		
		g7		
		B22		

## 4 DISCUSSION

Human tracts and traditional fermented food are important sources for screening lactic acid bacteria (LAB) with probiotic potential (Boricha et al., 2019; Masco et al., 2007; Qu et al., 2018). Previous researches showed that *Lactobacillus* strains have better tolerance to harsh environment than other kinds of lactic acid bacteria (LAB), especially low pH. Therefore, many *Lactobacillus* strains can be screened from traditional fermented food and human intestines (Chervinets et al., 2018; Devirgiliis et al., 2009). However, few studies compared the characteristics of *Lactobacillus* from different sources and screened probiotic *Lactobacillus* via an effective and efficient method. In this study, 40 strains of *Lactobacillus* from human intestines and traditional fermented food were compared in physiological characteristics, antioxidant and antibacterial ability. It was found that there were significant differences between *Lactobacillus* from human intestinal tracts and traditional fermented food in bile salt tolerance, adhesion to Caco‐2 cells, and antioxidant capacity of fermentation broth (*p* < .05). The PCA indicated human strains had better abilities and four probiotic strains CS39, CS128, CS01, and CS1301, had been selected for further application based on the heat‐map analysis with cluster analysis.

Tolerance to simulated digestion, especially low pH and bile salt, is the first step in the study of probiotic *Lactobacillus* (Klaenhammer & Kullen, 1999). In this study, the acid and bile salt tolerance of 40 strains from different sources were tested. The results showed that the tolerance of the strains to acid and bile salt was strain specific, which was consistent with the previous study (Bazireh et al., 2020). All the strains could maintain a survival rate of 10% at pH 3.0. However, Mulaw et al. (2019) found that only 16.07% of the strains from traditional Ethiopian fermented Teff injera dough, Ergo, and Kocho products could survive for 3 h at pH 3.0. In the present study, there was no significant difference between human and nonhuman strains at pH 2.0, 3.0 and 0.1% bile salt (*p* > .05). It may be that pickle, spicy sauce, and pickled cucumber provide a high osmotic pressure environment for the strains making the strains produce protective molecules (mainly proteins) to protect cells from damage (Mercedes Palomino et al., 2016). Thus, the nonhuman strains in this study had better tolerance. However, for 0.3% bile salt, the strains from human had better tolerance. The previous study found that low pH value of the growth environment would improve its resistant mechanism (Wang, Cui, & Qu, 2018). *Lactobacillus* strains isolated from human intestine must have experienced the adverse environment of human digestion, so they may have better tolerance. Different from the strains in traditional fermented food, human strains suffered from the gastric acid, intestinal juice, and bile salt, leading to greater adaptability and tolerance.

In addition to the acid and bile salt tolerance, the adhesion to intestinal tract is the premise of probiotic function and would help to promote immunomodulatory effects, as well as stimulate gut barrier and metabolic functions (Monteagudo‐Mera et al., 2019). The 40 strains in this study could adhere to Caco‐2 cells, enabling the strains to confer their probiotic functions before the gastrointestinal peristalsis removes the organisms (Byakika et al., 2019). However, different strains have different adhesion abilities, which are consistent with those given in a previous study (Lahteinen et al., 2010). The adhesion ability of isolated bacteria depends on the strain and is highly variable. However, *t* test on the adhesion ability of 40 strains of *Lactobacillus* showed that the adhesion ability of human strains was significantly higher than that of nonhuman strains (*p* < .05) (Figure 3). Chauviere et al. (1992) shared the same result that strains isolated from human have higher adhesion to intestinal cell lines. Human strains in this study were selected from the fecal samples of longevity elderly, indicating their ability to adhere to the gut in vivo. Therefore, the auto‐aggregation, hydrophobicity, and the ability to adhere to Caco‐2 cells of human strains were significantly higher than those of nonhuman (*p* < .05).

Oxidation is a major cause of many diseases, such as cardiovascular disease, inflammatory disease, cerebrovascular disease, degenerative disease, aging, and cancer (Grajek et al., 2005; Vasquez et al., 2019). Antioxidant capacity is one of the main probiotic properties (Wang et al., 2017). The total antioxidant capacity of 40 strains of *Lactobacillus* and their fermentation broths was tested. The results showed that the antioxidant capacity of the fermentation broth was much stronger than that of the strain itself, sharing the same results with those of the previous study (Shen et al., 2011). It may be that the metabolites, such as exopolysaccharides (Polak‐Berecka et al., 2013) and organic acids (Wu et al., 2021), are enriched in the fermentation broth making the fermentation broth have strong antioxidant capacity (Vasquez et al., 2019). In addition, it was found that human *Lactobacillus* strains had higher antioxidant capacity of fermentation broth (*p* < .01) and there was no significant difference in antioxidant capacity of strains (Figure 4), indicating that the metabolites of different strains were different.

Principal component analysis (PCA) and heat‐map analysis give a good overview of massive data and have been widely used in the visualization of metagenomic (Wintermans et al., 2015), meta‐transcriptomic (Mansfeldt et al., 2014), meta‐proteomic and metabolomics data (Vahedi et al., 2018). Divide the strains into some groups based on the probiotic properties and some studies have used this method to select candidate probiotic strains. In the present study, most strains were divided into two groups based on some properties via PCA while the classification was not completely clear‐cut, indicating that the strains from different sources had difference in properties but properties were based on specific strains. More interesting, significant difference has been found between scores of human and nonhuman strains (*p* < .05), meaning human tracts are a good source for isolating probiotic strains. The results of the heat‐map and unsupervised clustering algorithm analysis showed that four human strains and LGG were clustered into the same cluster, indicating that four strains, CS128, CS39, CS01, and CS1301, had better probiotic properties in 40 strains. The heat‐map analysis and the unsupervised clustering algorithm analysis shared the same result indicating their efficiency and scientificalness.

## 5 CONCLUSION

The PCA results showed that human strains have better probiotic properties. And four strains, CS128, CS39, CS01, and CS1301, from human tracts had been isolated for future application in functional foods based on their probiotic properties via the unsupervised clustering algorithm and heat‐map analysis.

## FUNDING INFORMATION

This study was financially supported by the National Natural Science Foundation of China (31972094), National Key Research and Development Program of China (2019YFF0217602), National Natural Science Foundation of the Jiangsu Higher Education Institutions of China (19KJA140004), Major Science and Technology Application Demonstration Program of Chengdu (2019‐YF09‐00055‐SN), and Science and Technology Achievements Transformation Demonstration Project in Sichuan (2018CC0147) and (XZ‐SZ202042). Postgraduate Research & Practice Innovation Program of Jiangsu Province (KYCX22_3500).

## CONFLICT OF INTEREST

The authors declare that they have no conflict of interest.

## References

[fsn33018-bib-0001] Agarbati, A. , Marini, E. , Galli, E. , Canonico, L. , Ciani, M. , & Comitini, F. (2021). Characterization of wild yeasts isolated from artisan dairies in the Marche region, Italy, for selection of promising functional starters. LWT‐Food Science and Technology, 139, 1–8. 10.1016/j.lwt.2020.110531

[fsn33018-bib-0002] Bazireh, H. , Shariati, P. , Jamalkandi, S. A. , Ahmadi, A. , & Boroumand, M. A. (2020). Isolation of novel probiotic *Lactobacillus* and *Enterococcus* strains from human salivary and fecal sources. Frontiers in Microbiology, 11, 597946–597958. 10.3389/fmicb.2020.597946 33343539 PMC7746552

[fsn33018-bib-0003] Boricha, A. A. , Shekh, S. L. , Pithva, S. P. , Ambalam, P. S. , & Vyas, B. R. M. (2019). In vitro evaluation of probiotic properties of *Lactobacillus* species of food and human origin. LWT‐Food Science and Technology, 106, 201–208. 10.1016/j.lwt.2019.02.021

[fsn33018-bib-0004] Byakika, S. , Mukisa, I. M. , Byaruhanga, Y. B. , & Muyanja, C. (2019). A review of criteria and methods for evaluating the probiotic potential of microorganisms. Food Reviews International, 35(5), 427–466. 10.1080/87559129.2019.1584815

[fsn33018-bib-0005] Chauviere, G. , Coconnier, M. H. , Kerneis, S. , Fourniat, J. , & Servin, A. L. (1992). Adhesion of human *Lactobacillus acidophilus* strain LB to human enterocyte‐like Caco‐2 cells. Journal of General Microbiology, 138(Pt 8), 1689–1696. 10.1099/00221287-138-8-1689 1527509

[fsn33018-bib-0006] Chervinets, Y. , Chervinets, V. , Shenderov, B. , Belyaeva, E. , Troshin, A. , Lebedev, S. , & Danilenko, V. (2018). Adaptation and probiotic potential of lactobacilli, isolated from the oral cavity and intestines of healthy people. Probiotics and Antimicrobial Proteins, 10(1), 22–33. 10.1007/s12602-017-9348-9 29164486

[fsn33018-bib-0007] Damodharan, K. , Lee, Y. S. , Palaniyandi, S. A. , Yang, S. H. , & Suh, J.‐W. (2015). Preliminary probiotic and technological characterization of *Pediococcus pentosaceus* strain KID7 and in vivo assessment of its cholesterol‐lowering activity. Frontiers in Microbiology, 6, 768–782. 10.3389/fmicb.2015.00768 26300852 PMC4523826

[fsn33018-bib-0008] Devirgiliis, C. , Coppola, D. , Barile, S. , Colonna, B. , & Perozzi, G. (2009). Characterization of the Tn916 conjugative transposon in a food‐borne strain of *Lactobacillus paracasei* . Applied and Environmental Microbiology, 75(12), 3866–3871. 10.1128/aem.00589-09 19395574 PMC2698359

[fsn33018-bib-0009] Ding, W. , Shi, C. , Chen, M. , Zhou, J. , Long, R. , & Guo, X. (2017). Screening for lactic acid bacteria in traditional fermented Tibetan yak milk and evaluating their probiotic and cholesterol‐lowering potentials in rats fed a high‐cholesterol diet. Journal of Functional Foods, 32, 324–332. 10.1016/j.jff.2017.03.021

[fsn33018-bib-0010] Domingos‐Lopes, M. F. P. , Stanton, C. , Ross, R. P. , & Silva, C. C. G. (2020). Histamine and cholesterol lowering abilities of lactic acid bacteria isolated from artisanal Pico cheese. Journal of Applied Microbiology, 129(6), 1428–1440. 10.1111/jam.14733 32500572

[fsn33018-bib-0011] Ferreira, C. L. , Grzeskowiak, L. , Carmen Collado, M. , & Salminen, S. (2011). In vitro evaluation of *Lactobacillus gasseri* strains of infant origin on adhesion and aggregation of specific pathogens. Journal of Food Protection, 74(9), 1482–1487. 10.4315/0362-028x.Jfp-11-074 21902917

[fsn33018-bib-0012] Grajek, W. , Olejnik, A. , & Sip, A. (2005). Probiotics, prebiotics and antioxidants as functional foods. Acta Biochimica Polonica, 52(3), 665–671. 16086074

[fsn33018-bib-0013] Hai, D. , Lu, Z. , Huang, X. , Lv, F. , & Bie, X. (2021). In vitro screening of chicken‐derived *Lactobacillus* strains that effectively inhibit salmonella colonization and adhesion. Food, 10(3), 569–583. 10.3390/foods10030569 PMC7998290 33803284

[fsn33018-bib-0014] Kang, W. , Pan, L. , Peng, C. , Dong, L. , Cao, S. , Cheng, H. , Gu, R. , Wang, J. , & Zhou, H. (2020). Isolation and characterization of lactic acid bacteria from human milk. Journal of Dairy Science, 103(11), 9980–9991. 10.3168/jds.2020-18704 32952010

[fsn33018-bib-0015] Klaenhammer, T. R. , & Kullen, M. J. (1999). Selection and design of probiotics. International Journal of Food Microbiology, 50(1–2), 45–57. 10.1016/s0168-1605(99)00076-8 10488843

[fsn33018-bib-0016] Kostelac, D. , Geric, M. , Gajski, G. , Markov, K. , Domijan, A. M. , Canak, I. , Jakopović, Z. , Sveteca, I. K. , Žunar, B. , & Frece, J. (2021). Lactic acid bacteria isolated from equid milk and their extracellular metabolites show great probiotic properties and anti‐inflammatory potential. International Dairy Journal, 112, 1–8. 10.1016/j.idairyj.2020.104828

[fsn33018-bib-0017] Krausova, G. , Hyrslova, I. , & Hynstova, I. (2019). In vitro evaluation of adhesion capacity, hydrophobicity, and auto‐aggregation of newly isolated potential probiotic strains. Fermentation, 5(4), 100. 10.3390/fermentation5040100

[fsn33018-bib-0018] Lahteinen, T. , Malinen, E. , Koort, J. M. K. , Mertaniemi‐Hannus, U. , Hankimo, T. , Karikoski, N. , Pakkanen, S. , Laine, H. , Sillanpaa, H. , Soderholm, H. , & Palva, A. (2010). Probiotic properties of *Lactobacillus* isolates originating from porcine intestine and feces. Anaerobe, 16(3), 293–300. 10.1016/j.anaerobe.2009.08.002 19695336

[fsn33018-bib-0019] Leandro, E. D. S. , Ginani, V. C. , De Alencar, E. R. , Pereira, O. G. , Paes Rose, E. C. , Martins do Vale, H. M. , Pratesi, R. , Machado Hecht, M. , Hermes Cavalcanti, M. , & Stéfany Oliveira Tavares, C. (2021). Isolation, identification, and screening of lactic acid bacteria with probiotic potential in silage of different species of forage plants, cocoa beans, and artisanal salami. Probiotics and Antimicrobial Proteins, 13(1), 173–186. 10.1007/s12602-020-09679-y 32601953

[fsn33018-bib-0020] Lee, Y. K. , Ho, P. S. , Low, C. S. , Arvilommi, H. , & Salminen, S. (2004). Permanent colonization by *Lactobacillus casei* is hindered by the low rate of cell division in mouse gut. Applied and Environmental Microbiology, 70(2), 670–674. 10.1128/aem.70.2.670-674.2004 14766540 PMC348792

[fsn33018-bib-0021] Liu, Z. , Jiang, Z. , Zhou, K. , Li, P. , Liu, G. , & Zhang, B. (2007). Screening of bifidobacteria with acquired tolerance to human gastrointestinal tract. Anaerobe, 13(5–6), 215–219. 10.1016/j.anaerobe.2007.05.002 17646115

[fsn33018-bib-0022] Mallappa, R. H. , Singh, D. K. , Rokana, N. , Pradhan, D. , Batish, V. K. , & Grover, S. (2019). Screening and selection of probiotic *Lactobacillus* strains of Indian gut origin based on assessment of desired probiotic attributes combined with principal component and heatmap analysis. LWT‐Food Science and Technology, 105, 272–281. 10.1016/j.lwt.2019.02.002

[fsn33018-bib-0023] Mansfeldt, C. B. , Rowe, A. R. , Heavner, G. L. W. , Zinder, S. H. , & Richardson, R. E. (2014). Meta‐analyses of *Dehalococcoides mccartyi* strain 195 transcriptomic profiles identify a respiration rate‐related gene expression transition point and interoperon recruitment of a key oxidoreductase subunit. Applied and Environmental Microbiology, 80(19), 6062–6072. 10.1128/aem.02130-14 25063656 PMC4178663

[fsn33018-bib-0024] Maresca, D. , Zotta, T. , & Mauriello, G. (2018). Adaptation to aerobic environment of *Lactobacillus johnsonii*/*gasseri* strains. Frontiers in Microbiology, 9(157), 1–11. 10.3389/fmicb.2018.00157 29479342 PMC5811513

[fsn33018-bib-0025] Masco, L. , Crockaert, C. , Van Hoorde, K. , Swings, J. , & Huys, G. (2007). In vitro assessment of the gastrointestinal transit tolerance of taxonomic reference strains from human origin and probiotic product isolates of *Bifidobacterium* . Journal of Dairy Science, 90(8), 3572–3578. 10.3168/jds.2006-548 17638965

[fsn33018-bib-0026] Mbye, M. , Baig, M. A. , AbuQamar, S. F. , El‐Tarabily, K. A. , Obaid, R. S. , Osaili, T. M. , Al‐Nabulsi, A. A. , Turner, M. S. , Shah, N. P. , & Ayyash, M. M. (2020). Updates on understanding of probiotic lactic acid bacteria responses to environmental stresses and highlights on proteomic analyses. Comprehensive Reviews in Food Science and Food Safety, 19(3), 1110–1124. 10.1111/1541-4337.12554 33331686

[fsn33018-bib-0027] Mercedes Palomino, M. , Waehner, P. M. , Fina Martin, J. , Ojeda, P. , Malone, L. , Sanchez Rivas, C. , Prado Acosta, M. , Allievi, M. C. , & Ruzal, S. M. (2016). Influence of osmotic stress on the profile and gene expression of surface layer proteins in *Lactobacillus acidophilus* ATCC 4356. Applied Microbiology and Biotechnology, 100(19), 8475–8484. 10.1007/s00253-016-7698-y 27376794

[fsn33018-bib-0028] Monteagudo‐Mera, A. , Rastall, R. A. , Gibson, G. R. , Charalampopoulos, D. , & Chatzifragkou, A. (2019). Adhesion mechanisms mediated by probiotics and prebiotics and their potential impact on human health. Applied Microbiology and Biotechnology, 103(16), 6463–6472. 10.1007/s00253-019-09978-7 31267231 PMC6667406

[fsn33018-bib-0029] Mulaw, G. , Tessema, T. S. , Muleta, D. , & Tesfaye, A. (2019). In vitro evaluation of probiotic properties of lactic acid bacteria isolated from some traditionally fermented Ethiopian food products. International Journal of Microbiology, 2019, 7179514. 10.1155/2020/6401356 31534458 PMC6732631

[fsn33018-bib-0030] Nami, Y. , Panahi, B. , Jalaly, H. M. , Bakhshayesh, R. V. , & Hejazi, M. A. (2020). Application of unsupervised clustering algorithm and heat‐map analysis for selection of lactic acid bacteria isolated from dairy samples based on desired probiotic properties. LWT‐Food Science and Technology, 118, 108839–108945. 10.1016/j.lwt.2019.108839

[fsn33018-bib-0031] Panahi, B. , Frahadian, M. , Dums, J. T. , & Hejazi, M. A. (2019). Integration of cross species RNA‐seq meta‐analysis and machine‐learning models identifies the most important salt stress‐responsive pathways in microalga *Dunaliella* . Frontiers in Genetics, 10, 752–764. 10.3389/fgene.2019.00752 31555319 PMC6727038

[fsn33018-bib-0032] Panahi, B. , Mohammadi, S. , Ruzicka, K. , Holaso, H. , & Mehrjerdi, M. (2019). Genome‐wide identification and co‐expression network analysis of nuclear factor‐Y in barley revealed potential functions in salt stress. Physiology and Molecular Biology of Plants, 25(2), 485–495. 10.1007/s12298-018-00637-1 30956430 PMC6419857

[fsn33018-bib-0033] Panahi, B. , Mohammadi, S. A. , & Doulati‐Baneh, H. (2020). Characterization of Iranian grapevine cultivars using machine learning models. Proceedings of the Indian National Science Academy Part B Biological Sciences, 90(3), 615–621. 10.1007/s40011-019-01131-8

[fsn33018-bib-0034] Park, Y. S. , Lee, J. Y. , Kim, Y. S. , & Shin, D. H. (2002). Isolation and characterization of lactic acid bacteria from feces of newborn baby and from dongchimi. Journal of Agricultural and Food Chemistry, 50(9), 2531–2536. 10.1021/jf011174i 11958617

[fsn33018-bib-0035] Polak‐Berecka, M. , Wasko, A. , Szwajgier, D. , & Choma, A. (2013). Bifidogenic and antioxidant activity of exopolysaccharides produced by *Lactobacillus rhamnosus* E/N cultivated on different carbon sources. Polish Journal of Microbiology, 62(2), 181–188. 10.33073/pjm-2013-023 24053021

[fsn33018-bib-0036] Qian, Y. , Zhang, J. , Zhou, X. , Yi, R. , Mu, J. , Long, X. , Pan, Y. , Zhao, X. , & Liu, W. (2018). *Lactobacillus plantarum* CQPC11 isolated from Sichuan pickled cabbages antagonizes d‐galactose‐induced oxidation and aging in mice. Molecules, 23(11), 3026–3043. 10.3390/molecules23113026 30463304 PMC6278364

[fsn33018-bib-0037] Qu, H. , Yu, H. , Gu, R. , Chen, D. , Chen, X. , Huang, Y. , Xi, W. , & Huang, Y. (2018). Proteomics for studying the effects of *L. rhamnosus* LV108 against non‐alcoholic fatty liver disease in rats. RSC Advances, 8(67), 38517–38528. 10.1039/c8ra06771f 35559112 PMC9090571

[fsn33018-bib-0038] Sharma, K. , Pooranachithra, M. , Balamurugan, K. , & Goel, G. (2019). Multivariate analysis of increase in life span of *Caenorhabditis elegans* through intestinal colonization by indigenous probiotic strains. Probiotics and Antimicrobial Proteins, 11(3), 865–873. 10.1007/s12602-018-9420-0 29717419

[fsn33018-bib-0039] Shen, Q. , Shang, N. , & Li, P. (2011). In vitro and in vivo antioxidant activity of *Bifidobacterium animalis* 01 isolated from centenarians. Current Microbiology, 62(4), 1097–1103. 10.1007/s00284-010-9827-7 21132298

[fsn33018-bib-0040] Shobharani, P. , & Agrawal, R. (2011). A potent probiotic strain from Cheddar cheese. Indian Journal of Microbiology, 51(3), 251–258. 10.1007/s12088-011-0072-y 22753999 PMC3209920

[fsn33018-bib-0041] Shokryazdan, P. , Faseleh Jahromi, M. , Liang, J. B. , & Ho, Y. W. (2017). Probiotics: From isolation to application. Journal of the American College of Nutrition, 36(8), 666–676. 10.1080/07315724.2017.1337529 28937854

[fsn33018-bib-0042] Torriani, S. , Felis, G. E. , & Dellaglio, F. (2001). Differentiation of *Lactobacillus plantarum*, *L. pentosus*, and *L. paraplantarum* by recA gene sequence analysis and multiplex PCR assay with recA gene‐derived primers. Applied and Environmental Microbiology, 67(8), 3450–3454. 10.1128/aem.67.8.3450-3454.2001 11472918 PMC93042

[fsn33018-bib-0043] Vahedi, M. , Kabiri, M. , Salami, S. A. , Rezadoost, H. , Mirzaie, M. , & Kanani, M. R. (2018). Quantitative HPLC‐based metabolomics of some Iranian saffron (*Crocus sativus* L.) accessions. Industrial Crops and Products, 118, 26–29. 10.1016/j.indcrop.2018.03.024

[fsn33018-bib-0044] Vasiee, A. , Falah, F. , Behbahani, B. A. , & Tabatabaee‐yazdi, F. (2020). Probiotic characterization of *Pediococcus* strains isolated from Iranian cereal‐dairy fermented product: Interaction with pathogenic bacteria and the enteric cell line Caco‐2. Journal of Bioscience and Bioengineering, 130(5), 471–479. 10.1016/j.jbiosc.2020.07.002 32753308

[fsn33018-bib-0045] Vasquez, E. C. , Pereira, T. M. C. , Peotta, V. A. , Baldo, M. P. , & Campos‐Toimil, M. (2019). Probiotics as beneficial dietary supplements to prevent and treat cardiovascular diseases: Uncovering their impact on oxidative stress. Oxidative Medicine and Cellular Longevity, 33(3), 339–343. 10.1155/2019/3086270 PMC6530239 31205584

[fsn33018-bib-0046] Wang, C. , Cui, Y. , & Qu, X. (2018). Mechanisms and improvement of acid resistance in lactic acid bacteria. Archives of Microbiology, 200(2), 195–201. 10.1007/s00203-017-1446-2 29075866

[fsn33018-bib-0047] Wang, Q. , Gao, Q. , Gao, X. , & Nie, F. (2018). L(2, p)‐norm based PCA for image recognition. IEEE Transactions on Image Processing, 27(3), 1336–1346. 10.1109/tip.2017.2777184 29989986

[fsn33018-bib-0048] Wang, W. , Liu, W. , & Chu, W. (2020). Isolation and preliminary screening of potentially probiotic *Weissella confusa* strains from healthy human feces by culturomics. Microbial Pathogenesis, 147, 3026–3043. 10.1016/j.micpath.2020.104356 32610159

[fsn33018-bib-0049] Wang, Y. , Wu, Y. P. , Wang, Y. Y. , Xu, H. , Mei, X. Q. , Yu, D. Y. , Wang, Y. , & Li, W. F. (2017). Antioxidant properties of probiotic bacteria. Nutrients, 9(5), 521–536. 10.3390/nu9050521 28534820 PMC5452251

[fsn33018-bib-0050] Wintermans, B. , Brandt, B. , Vandenbroucke‐Grauls, C. , & Budding, A. (2015). TreeSeq, a fast and intuitive tool for analysis of whole genome and metagenomic sequence data. PLoS One, 10(5), 1–11. 10.1371/journal.pone.0123851 PMC4416914 25933115

[fsn33018-bib-0051] Wu, R. , Zhang, W. , Sun, T. , Wu, J. , Yue, X. , Meng, H. , & Zhang, H. (2011). Proteomic analysis of responses of a new probiotic bacterium *Lactobacillus casei* Zhang to low acid stress. International Journal of Food Microbiology, 147(3), 181–187. 10.1016/j.ijfoodmicro.2011.04.003 21561676

[fsn33018-bib-0052] Wu, Y. , Li, S. , Tao, Y. , Li, D. , Han, Y. , Show, P. L. , Wen, G. , & Zhou, J. (2021). Fermentation of blueberry and blackberry juices using *Lactobacillus plantarum*, *Streptococcus thermophilus* and *Bifidobacterium bifidum*: Growth of probiotics, metabolism of phenolics, antioxidant capacity in vitro and sensory evaluation. Food Chemistry, 348, 1–16. 10.1016/j.foodchem.2021.129083 33517000

